# Voluntary interruption of pregnancy using hysteroresectoscopy: a mini-series of 15 cases

**DOI:** 10.3389/fgwh.2026.1847030

**Published:** 2026-07-22

**Authors:** Ophélie Revret, Louis Van Hees, Charlotte Maillard, Mathieu Luyckx, Amandine Gerday

**Affiliations:** 1 Department of Gynaecology and Andrology, Cliniques Universitaires Saint-Luc, Brussels, Belgium; 2 Institut De Duve, Université Catholique de Louvain, Brussels, Belgium; 3 Institut Roi Albert 2- IRA 2, Cliniques Universitaires Saint-Luc, Brussels, Belgium

**Keywords:** complications, dilatation and curettage, hysteroscopic resection, surgical abortion, synechiae, voluntary interruption of pregnancy

## Abstract

**Objective:**

Although surgical techniques for later interruption have advanced since the legalisation of voluntary interruption of pregnancy (VIP), dilatation and curettage via aspiration (D&C) remains the conventional approach. Hysteroresectoscopy (HsR) presents a potential alternative. This study aims to evaluate the feasibility of using HsR to perform VIP in cases in the early first trimester.

**Design:**

A case series involving patients treated for VIP in our department.

**Patients:**

Women seeking VIP between six and ten weeks of amenorrhoea.

**Intervention:**

Hysteroscopic resection following cervical dilatation, performed as an outpatient procedure under general or spinal anaesthesia.

**Results:**

Between January 2021 and January 2026, 15 patients underwent VIP via HsR at our institution. One suspected case of uterine perforation was reported, and one patient experienced retained products of conception, which were managed with hysteroscopy performed in consultation without anesthesia. Additionally, 46,6% of patients received simultaneous contraception implantation.

**Conclusion:**

This mini-series suggests that HsR is a feasible technique for first trimester VIP, ideally applicable to pregnancies of up to nine of amenorrhoea. Fertility gains are expected from the minimal endometrial damage permitted by the technique. The findings are limited by the small sample size, making it difficult to draw definitive conclusions regarding the safety or efficacy of hysteroscopy compared with medical management or D&C. Although hysteroscopy appears to be a safe and promising approach, larger prospective studies are needed to confirm these findings and better compare outcomes with those of D&C.

## Introduction

In Belgium, voluntary interruption of pregnancy (VIP) has been legal since March 1990. It is governed by regulations including a mandatory six-day reflection period before the procedure, and it is permitted until 14 weeks of gestation (1). With an estimated global incidence of approximately 35 per 1,000 women, VIP is a highly prevalent procedure (2, 3). Although advances in medical management have led to the development of medical evacuation techniques; however, D&C remains the standard surgical approach.

Despite the higher rate of incomplete evacuation associated with the medical approach, the global trend is to prefer medical methods since the surgical approach can present potential complications, that may impede future fertility in younger patients (4–6). Despite encouraging results from recent studies exploring the use of hyaluronic acid to mitigate risks, uterine synechiae following surgical interventions remain a concern (7).

The initial rationale for adopting hysteroscopic management in this context was to provide an alternative approach for patients with significant comorbidities, including solid-organ transplant, coagulation disorders, or those receiving anticoagulant or other treatments potentially increasing the risk of bleeding or perioperative complications during medical management of expulsion, whilst avoiding conventional suction curettage for small gestational sacs. In these higher-risk clinical situations, hysteroscopy was considered as a potentially safer and more controlled minimally invasive option.

Based on our experience of using HsR to treat missed abortions and molar pregnancies (8, 9), we suggested that HsR could be a less invasive alternative, maintaining the benefits to D&C, while still offering the same benefits, such as the ability to perform simultaneous contraception implantation and expedited intervention, as well as lower rates of retained product of conception (RPOC). Unlike aspiration-based procedures that subject the uterine walls to non-specific treatment, HsR enables targeted dissection of the gestational sac, while preserving endometrial integrity which could enhance future fertility.

To our knowledge, this is the first report on the use of this technique for VIP. Our goal is to demonstrate its feasibility, evaluate the safety of the surgery, and discuss its associated benefits and limitations.

## Material and method

Following approval from our ethics committee (reference number: 2025/17FEV/075), we conducted a retrospective review of clinical data from Cliniques Universitaires Saint-Luc in Brussels, from 2021 to 2026. We identified 15 women who underwent VIP via HsR during this period. Data were extracted occurred from electronic medical records, focusing on demographics, surgical details, postoperative complications, and follow-up. The primary outcomes were intraoperative complications (e.g., estimated blood loss >1000 mL, conversion to D&C, and uterine perforation), the secondary outcomes focused on post-procedure RPOC incidence.

### Operative technique

**Figure 1:**
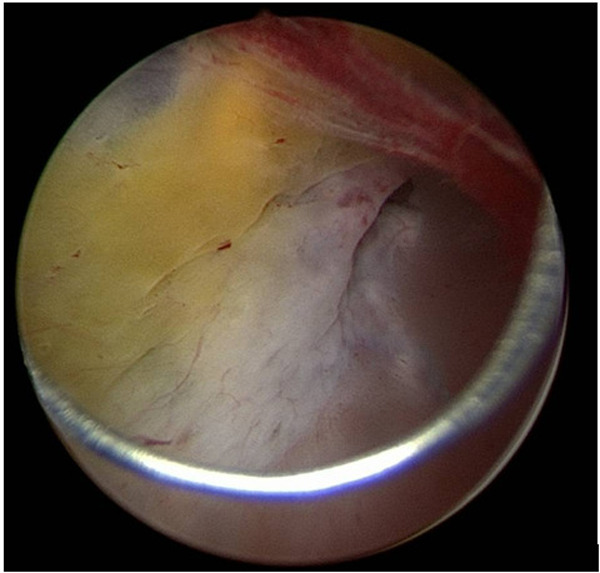
Intra-operative view with intact gestational sac.

**Figure 2:**
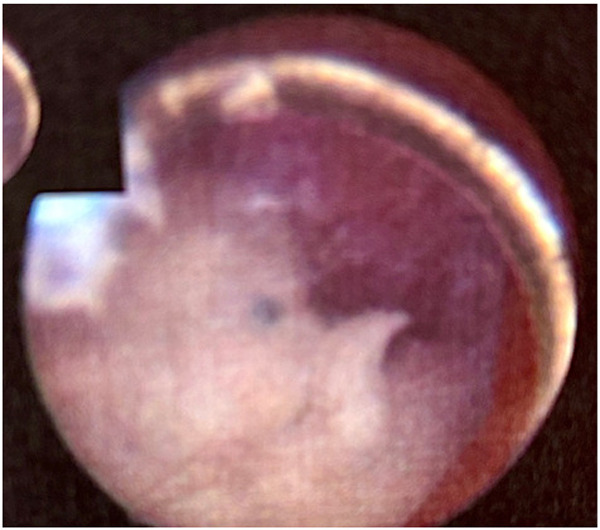
Intra-operative view showing with the embryo.

Ambulatory surgery was performed under general or spinal anaesthesia. Cervical dilation was carried out to a diameter of Hegar No. 10. The hystero-resectoscope was introduced into the uterine cavity, to allow identification of the gestational sac and, in some cases the embryo (Figures 1, 2). Trophobastic tissue was removed using a mechanical loop without the application of electric energy. The procedure was performed with a continuous flow of glycine or saline to distend the uterus. Glycine was used for the first 11 patients, and normal saline was used for the last four. The reason for this change is simply that our surgical equipment for operative hysteroscopy was upgraded to us bipolar energy.

After completely resecting the trophoblastic tissue, the hystero-resectoscope was removed, and forceps were inserted to retrieve any remaining material. The hysteroscope was then reintroduced to confirm that the uterine cavity was empty. All retrieved tissue was sent for histopathological analysis.

During the procedure, glycine/saline was collected in a plastic bag positioned under the patient. Postoperatively, we assessed for any potential absorption of glycine/saline. If significant absorption of glycine exceeding 1000 mL occurred, we monitored serum electrolyte levels and recommended the administration of a diuretic (furosemide).

In cases of persistent blood loss, three 200 mg Misoprostol tablets were administered rectally.

This surgical technique was consistently performed by two surgeons throughout the study period.

## Results

Between January 2021 and January 2026, 15 patients underwent VIP via operative hysteroscopy. At the time of surgery, the mean gestational age was 8.15 ± 1.18 weeks, ranging from 6 weeks and 0 days to 9 weeks and 4 days. The average patients age was 34 years, with most patients being multiparous (*p* = 0.018) (Table 1).

**Table 1:** Demographic data.

Demographic data	Patients (*n* = 15)
Age (years; mean ± SD)	34,07 ± 5,78
Gravidity (*n*; mean ± SD)	3,93 ± 1,28
Parity (*n*; mean ± SD)	2,27 ± 1,16
Gestational age at intervention (weeks ± SD)	8,15 ± 1,18

The mean operative time was 25.7 ± 9.8 min (range 14–40 min), with one of the longest procedures involving simultaneous tubal sterilisation.

A single intraoperative event involved a suspected uterine perforation based on the appearance of yellow tissue suggestive of fat, although no uterine defect was confirmed on complete hysteroscopic inspection, indicating a suspected rather than confirmed perforation, which should be interpreted cautiously given the small sample size (Table 2).

**Table 2:** Operative data.

Operative data	Patients (*n* = 15)
Intervention time (minutes; mean ± SD)	25,7 ± 9,8
REsorption glycine >500 mL (*n*, %)	5, 45%
REsorption glycine >1000 mL (*n*, %)	0
Use of Misoprostol (*n*, %)	2, 13,3%
Complication (*n*, %)	1, 6,6%
Conversion (*n*, %)	0

The mean glycine absorption was 337 ± 331 mL, with five patients (45%) experiencing absorption exceeding 500 mL, while none exceeded 1000 mL. The maximum glycine absorption was 900 mL, which was observed in a patient with suspected uterine perforation. No complication were related to glycine absorption. For the final four patients, normal saline was used for the uterine distension. The mean saline absorption was 1,000 ± 530 mL without patient absorbing more than 2,500 mL. The maximum glycine absorption recorded was 1,400 mL. No complications related to glycine absorption observed.

Misoprostol was administered to two patients intraoperatively in the setting of moderate bleeding to enhance uterine tone, rather than for cervical preparation or management of suspected retained products of conception, and its potential influence on postoperative outcomes cannot be fully excluded. However, no bleeding exceeding 500 mL was recorded and bleeding was classified as “minimal” in 12 patients (80%).

One patient also underwent the removal of a levonorgestrel-releasing intrauterine device (Mirena®) during the hysteroscopy.

One month postoperatively, a pelvic scan was performed on 12 out of 15 patients. In cases of suspected RPOC, diagnostic hysteroscopy as performed. Two patients underwent this procedure, one for persistent RPOC, which was confirmed and successfully treated by hysteroscopic resection (performed in consultation without anesthesia), and another for persistent metrorrhagia in a complex clinical context involving chronic hemodialysis and future intrauterine device insertion, in whom no retained tissue was identified. Given the limited cohort size, these findings should be interpreted with caution, as the occurrence of a single event may substantially influence the apparent complication rate.

Most patients (86.6%) were discharged with a contraceptive method, with seven patients opting for long-acting reversible contraception. Two patients received follow-up care from external gynaecologists, and one patient was lost to follow-up (Table 3).

**Table 3:** Follow-up data.

Follow-up data	Patients
RPOC (*n*, %)	1, 7,1% (*N* = 14)
Contraception implantation (*n*, %)	7, 46,6% (*N* = 15)

## Discussion

When a surgical VIP is indicated, dilation and curettage (D&C) is the conventional procedure. This small series presents an alternative that appears to be a feasible and relatively straightforward option for pregnancies up to nine weeks of amenorrhoea (Table 4). The mean operative time of 25.7 min aligns with findings from our previous series, which used a similar operative technique for different indications such as failed abortion (20 min) and molar pregnancies (29 min) (7, 8).

**Table 4:** Detailed data.

Demographics data					Operatives data			
Age	Gravidity Parity	Gestational Age (weeks of amenorrhea)	Gestational Sac Size (mm)	Embryo Size (mm)	Operative Time (min)	Distension Fluid Absorption (mL)	Bleeding	Complications
36	G3P2	6 + 6/7	25	8	36	50 glycine	minimal	/
40	G5P2	6 + 0/7	7	0	14	50 glycine	minimal	/
32	G3P2	8 + 2/7	/	11	23	750 glycine	minimal	retained products of conception
40	G4P3	8 + 3/7	41	13	15	0 glycine	minimal	/
32	G2P0	7 + 2/7	/	6.5	23	0 glycine	minimal	/
44	G7P5	8 + 4/7	/	7.5	12	0 glycine	minimal	/
27	G2P1	8 + 3/7	34	12	27	550 glycine	moderate	/
31	G4P2	9 + 4/7	51	20	23	500 glycine	minimal	/
34	G3P2	8 + 2/7	33	11	36	300 glycine	moderate	/
24	G4P2	8 + 5/7	39	13	17	700 glycine	minimal	/
34	G4P3	9 + 3/7	25	17	31	900 glycine	minimal	suspected uterine perforation
33	G4P1	6 + 0/7	10	0	16	250 saline	minimal	/
37	G5P3	9 + 1/7	35	16.4	38	1400 saline	moderate	/
26	G4P3	7 + 1/7	15	4.5	34	1000 saline	minimal	/
41	G5P3	8 + 3/7	27	11.9	40	1350 saline	minimal	/

However, surgery was reported as more challenging in more advanced pregnancies beyond 9 weeks due to the increased size of the uterine cavity, which made it difficult for the hysteroscope to reach the fundus. Using an XXL hysteroscope, typically employed for obese patients, could potentially address this issue in such cases. However, larger uterine cavities may increase the risk of bleeding and higher fluid absorption (9). The feasibility limit of this approach remains unclear, but it could be extended as we gain more experience in its application.

Several limitations of hysteroscopic management should also be acknowledged. Compared with conventional curettage, hysteroscopy is associated with higher procedural costs due to the need for specialised equipment. Additionally, operative time may be a limitation of the technique, particularly as it is highly dependent on the surgeon's expertise and the complexity of the case. Furthermore, there is currently no clear evidence to suggest that the risk of uterine perforation is significantly reduced compared with conventional curettage techniques.

One of the key objectives of this technique is to minimise potential damage to the uterine cavity, particularly in younger patients who may still wish to conceive in the future. A recent publication by Huchon et al. demonstrated no significant differences in future pregnancy outcomes between patients treated for failed abortion with hysteroscopic resection (HSR) and those treated with D&C (10). Nevertheless, this effect requires further validation in the failed abortion population before VIP can be expanded for this indication. Nevertheless, our team remains convinced that this technique could be an additional surgical technique to offer our patients in the context of pregnancy termination for well-defined indications. The visual control offered by the technique may reduce the rate of retained trophoblastic tissue, thereby lowering the need for secondary surgeries (11).

A recent study by Zhou et al. compared the outcomes of traditional ultrasound-guided VIP with those of direct uterine visualisation aspiration device. Their results showed significant advantages for the direct visualisation method, includindg lower postoperative pain and bleeding. Additionally, women in the direct visualisation group were more likely to resume normal menstruation in terms of both incidence and duration. These outcomes were likely due to more precise suction techniques (10).

While recent studies suggest that mechanical cervical dilation has no adverse effects on future obstetrical outcomes (12), our operative technique requires dilation up to Hegar size 10. In contrast, D&C procedures often use cannulas with smaller diameter, which necessitate less cervical dilation (14). Some D&C VIP protocols include cervical preparation with misoprostol, enabling the procedure to be performed with smaller cannulas (6–8 mm) without the need for mechanical dilation, since the cervix is already softened. The development of smaller-diameter hysteroscopic instruments may reduce the invasiveness of hysteroscopic pregnancy interruption procedures. Miniaturized hysteroscopes and resectoscopes with outer diameters around 5–6 mm could allow treatment with limited cervical dilation or, in selected cases, without mechanical dilation, thereby reducing cervical trauma. However, reducing instrument size may also have drawbacks, including decreased resection efficiency, longer operative times, reduced visualization, and a potentially increased risk of uterine perforation.

In addition, very early pregnancies (less than 8 weeks of gestation) may represent an attractive indication for small-caliber hysteroscopic techniques, as the volume of gestational tissue is limited and may be completely removed more easily.

As these questions were not studied here, further prospective studies are needed to assess feasibility, completeness of evacuation, operative time, safety, and the ability of this approach to preserve the benefits of direct hysteroscopic visualization.

This technique also enables a contraceptive device to be placed simultaneously. Furthermore, additional intrauterine procedures can be performed during the same operative session. For instance, one patient underwent the removal of a levonorgestrel-releasing intrauterine device (Mirena®) during hysteroscopy, without an increase in operative risk.

In addition to being used for voluntary termination of pregnancy, hysteroscopy has also been described as a potential therapeutic option for caesarean scar pregnancy in selected cases. Although this indication was not specifically investigated in the present study, operative hysteroscopy may represent an interesting minimally invasive approach for carefully selected patients, either alone or in combination with other therapeutic strategies (13).

As with other indications for operative hysteroscopy, saline should be used as the distension medium, particularly in pregnant patients, as they tend to absorb more fluid. Complications have been reported with absorption volumes exceeding 500 mL, although most guidelines recommend medical monitoring when more than 1,000 mL is absorbed (9).

The main limitation of this study is the small sample size, with only 15 patients were included. Consequently, the statistical power of the study remains limited, and potential selection bias cannot be excluded. Larger prospective studies are therefore required to confirm the reproducibility and generalizability of these findings.

Although operative hysteroscopic resection (HSR) cannot entirely replace D&C, it could be a valuable option for carefully selected patients. Given the limited sample size of the present study, we consider that it is not yet appropriate to establish definitive selection criteria based on our data. This will require confirmation in a larger prospective cohort. Nevertheless, based on the technical challenges encountered during the procedures, we suggest that hysteroscopic management should, at this stage, be used for pregnancy of less than 10 weeks’gestation where there is a potential contraindication to medical treatment (coagulation disorder, medical history, patient refusal of medical treatment,…).

The resources required to treat all VIPs via HSR are considerable. Therefore, this technique should be reserved for cases in wich the benefits of HSR, such as visual control and potentially reduced complications, outweigh those of D&C.

## Conclusions

HsR appears to be a feasible and relatively straightforward technique for VIP up to 9 weeks of amenorrhea in appropriately selected cases, although technical difficulties may arise beyond this gestational age. Within this feasibility framework, hysteroscopic management represents a promising minimally invasive alternative approach.

However, the present findings are limited by the small sample size, which precludes robust statistical conclusions and does not allow definitive assessment of comparative safety or efficacy versus medical management or D&C. In particular, a clear reduction in complication rates cannot be established based on the available data.

Nevertheless, the potential advantages of hysteroscopy, including minimal endometrial trauma due to direct visualisation and controlled dissection, may make it a valuable option in selected patients, particularly in cases with comorbidities or relative contraindications to curettage. Further larger prospective studies are required to better define its role, refine selection criteria, and confirm its safety profile and possible impact on fertility outcomes.

## Data Availability

The raw data supporting the conclusions of this article will be made available by the authors, without undue reservation.
